# Supplementary material for: Precise Targeting of Autoantigen-Specific B Cells in Lupus Nephritis with Chimeric Autoantibody Receptor T Cells
Source: Int J Mol Sci. 2024 Apr 11;25(8):4226. doi: 10.3390/ijms25084226 (PMC11050013; doi:10.3390/ijms25084226)
Supplement: Supplementary file 1 [file ijms-25-04226-s001.zip › ijms-2904840-supplementary.pdf]

Supporting Information

Precision targeting of autoantigen-specific B cells in LN with chimeric autoantibody receptor T cells

Cristina Solé [1*], Maria Royo[1], Sebastian Sandoval[1], Teresa Moliné[2], Alejandra Gabaldón[2], Josefina Cortés-Hernández[1]

[1] Rheumatology Research Group - Lupus Unit, Vall d'Hebrón University Hospital, Vall d'Hebrón Research Institute (VHIR), Universitat Autònoma de Barcelona (UAB), Barcelona, Spain.[2] Department of Pathology, Vall d'Hebrón University Hospital, Barcelona, Spain.

INDEX

1. SI Materials and Methods.. 3

2. References..5

3. Supplementary Figures 6

4. Supplementary Tables 12

1. SI Materials and Methods

Blood samples

Blood samples were taken and centrifuged at 3.000 rpm for 30 minutes at room temperature. After that, the layer containing PBMCs was collected using a pipette. Cells were washed twice with PBS and resuspended in complete RPMI media (RPMI, 10% FBS, 10% penicillin/streptomycin, Gibco, ThermoFisher Scientific, MA, USA). Serum was obtained using SST Serum separation tubes (BD Vacutainer, NYSE, USA) after centrifugation for 15 minutes at 1500 rpm room temperature. Samples were stored at -80º C for further use.

Isolation of primary human T cells

Primary human T cells were isolated from patient's PBMCs using "Dynabeads Un-touched Human T Cells Kit" (ThermoFisher Scientific, MA, USA) following manufacturer's instruction (more details in SI). Cells were cultured in RPMI1640, 10% FBS and 1% penicillin/streptomycin. Bulk T cells (CD4+ and CD8+) were stimulated with anti-CD3 and anti-CD28 beads (Dynabeads, Life Technologies, CA, USA) at a bead:cell ratio of 3:1. The culture medium was supplemented with human interleukin-2 (20ng/mL, ThermoFisher Scientific, MA, USA) for 24 hours.

Isolation and culture of primary human B cells

Primary human B cells from the study groups (anti-dsDNA$^{+/-}$ antibodies) were isolated from PBMCs samples using "Dynabeads Untouched Human B cells Kit" (Invitrogen, MA, USA) following manufacturer's instruction. B cells were cultured using RPMI-1640 medium (Gibco Thermofisher Scientific) sup-plemented with 10% (v/v) heat-inactivated fetal bovine serum (FBS) and 5% penicillin/streptomycin. To evaluate their capacity to produce antibodies, isolated B cells were platted on U-bottom 96-well plates at concentration of 1x105 and stimulated with INFα, IFNβ (50 ng/mL, Gibco, Thermofisher Scientific, MA, USA) or PBS (control conditions) for 6-48 hours. After that, culture medium was analysed by enzyme-linked immunosorbent assay (ELISA).

RNA extraction and RT-qPCR

Total RNA from isolated B or T cells was extracted using the miRNeasy® Mini Kit (Qi-agen, Hilden, Germany) according to manufacturer instructions. The complementary DNA (cDNA) was obtained from 250 ng of the purified RNA using the High-Capacity cDNA Reverse Transcription Kit (Applied Biosystems, MA, USA). qPCR was performed on a 7000 ABI Thermofisher (Applied Biosystems, MA, USA) using a TaqMan gene ex-pression assay (FAM dye labeled MGB probe (Applied Biosystems, MA, USA) and the gene-specific primers and probes are shown in Table S1 (more details in SI). After qPCR, relative expression was determined using the data from the real-time cycler and the ΔΔCT method. Differences in relative mRNA expression of target genes between con-trol and therapy groups were expressed as fold-changes

Kidney organoid culture from frozen stocks

Frozen kidney organoid stocks were purchased from Hubrecht Organoid Technology (Utrecht, The Netherlands, passage 4-6). We followed their confidential optimised protocols for their manipulation. The stock and organoid (1xKEM) medium was prepared following Hubrecht Organoid Technology instructions. We took a cryovial containing organoids from the liquid nitrogen tank and we transfer it rapidly into a 37ºC water bath by 2 minutes. We added 0,5mL of warm stock medium to the cryovial and afterwards, the solution was transferred into a 15mL plastic tube with 12mL of pre-warmed stock medium. We centrifuged the plastic tube 5 min at 700 rpm at 4ºC. We resuspended the organoid pellet with only 50ul of 70-80% matrigel / Cultrex

Basal Membrane Extracts (MG/BME). We planted small drops into pre-warmed 6 or 24-well cell culture plate to be left into a 37ºC incubator during 60 minutes for MG/BME to solidify. After that, we added 2mL or 500ul of 1Xkem medium with 10 µM Rho kinase inhibitor (RhoKi) to be transferred back to the incubator at 37ºC with 5% CO2. Medium was refreshed every 3 days and, after 7 days, kidney organoids were used to perform the corresponding experiments. We used 2mL or 500ul of Corning Cell Recovery Solution (Corning Inc., NY, USA) in order to disrupt kidney organoid and make easy their passing.

Detection of IgG autoantibodies by in-house ELISA assay

Serum anti-α-actinin, anti-histone1 and anti-heparan sulfate from SLE patients (N=10) were measured by ELISA following the methodology described by Cortés-Hernández J *et al* [63]. Human α–actinin, histone 1 and heparan sulfate (Merck Life Science, Spain) were used to coat ELISA plates at a concentration of 0.250 ug/mL in sodium bicarbonate buffer of pH9.6 for 1h. Plates were blocked for 1h with SuperBlock (ThermoFisher Scientific, MA, USA) and kept at 4ºC until use. Patient serum diluted 1:50 in SuperBlock containing 0.1% Tween 20 was added in triplicate to the ELISA plate, which also contained a standard dilution series of STD2 and QC1 in duplicate wells (a between-plate quality control sample). Following 2h incubation at room temperature on a plate shaker, the plates were washed thoroughly (x6 times) with phosphate-buffered saline (PBS) plus 0.1% Tween 20. Anti-human IgG conjugated to horseradish peroxidase (HRP) preabsorbed (Abcam, Cambridge, UK) diluted 1:5000 in SuperBlock was added (100 ml per well) and incubated for 2hours as described earlier. After washing as described, enzyme substrate 3,3',5,5'- Tetramethylbenzidine (TMB, Merck Life Science, Spain) was added, developed for 20 min at 37ºC, and the reaction was stopped with 0.5M H_2SO_4 .The plates were read at 450 nm, standard curves were plotted and values were assigned to samples. Positive samples were considered above 25UI/mL by using the mean +3 standard deviation of the healthy group control. The cut-off level of ELISA detection was established at 7UI/mL. To test ELISA methodology, a healthy control group was used (N=30), none of whom were positive. To perform the standard curve, we used a pool of healthy donor serum with 1/5, 1/10, 1/25, 1/50, 1/100, 1/250, 1/500 and 1/1000 concentration of commercial corresponding autoantibody. Quality control sample (QC1) was used for each ELISA plate and consisted of a pool of borderline positive samples (20-25 UI/mL, n=5).

2. References

[66] Cortés-Hernández J, Ordi-Ros J, Labrador M, Buján S, Balada E, Segarra A, Vilardell-Tarrés M. Antihistone and anti-double-stranded deoxyribonucleic acid antibodies are associated with renal disease in systemic lupus erythematosus. *Am J Med.* **2004**; 116: 165-73.

3. **Supplementary Figures**

Supplementary Figure S1. **Map of the CAAR expression vector used to transfect T cells.** Vector containing human protein sequence depend on the antigen target: alpha-actin, heparan sulphate, histone-1 or C1q. Amp R, ampicillin resistance gene; WPRE, Woodchuck Hepatitis Virus (WHP) Posttranscriptional Regulatory Element; LTR, Long Terminal Repeat; SV40, simian virus 40; hEF1a, Human elongation factor-1 alpha (EF-1 alpha).

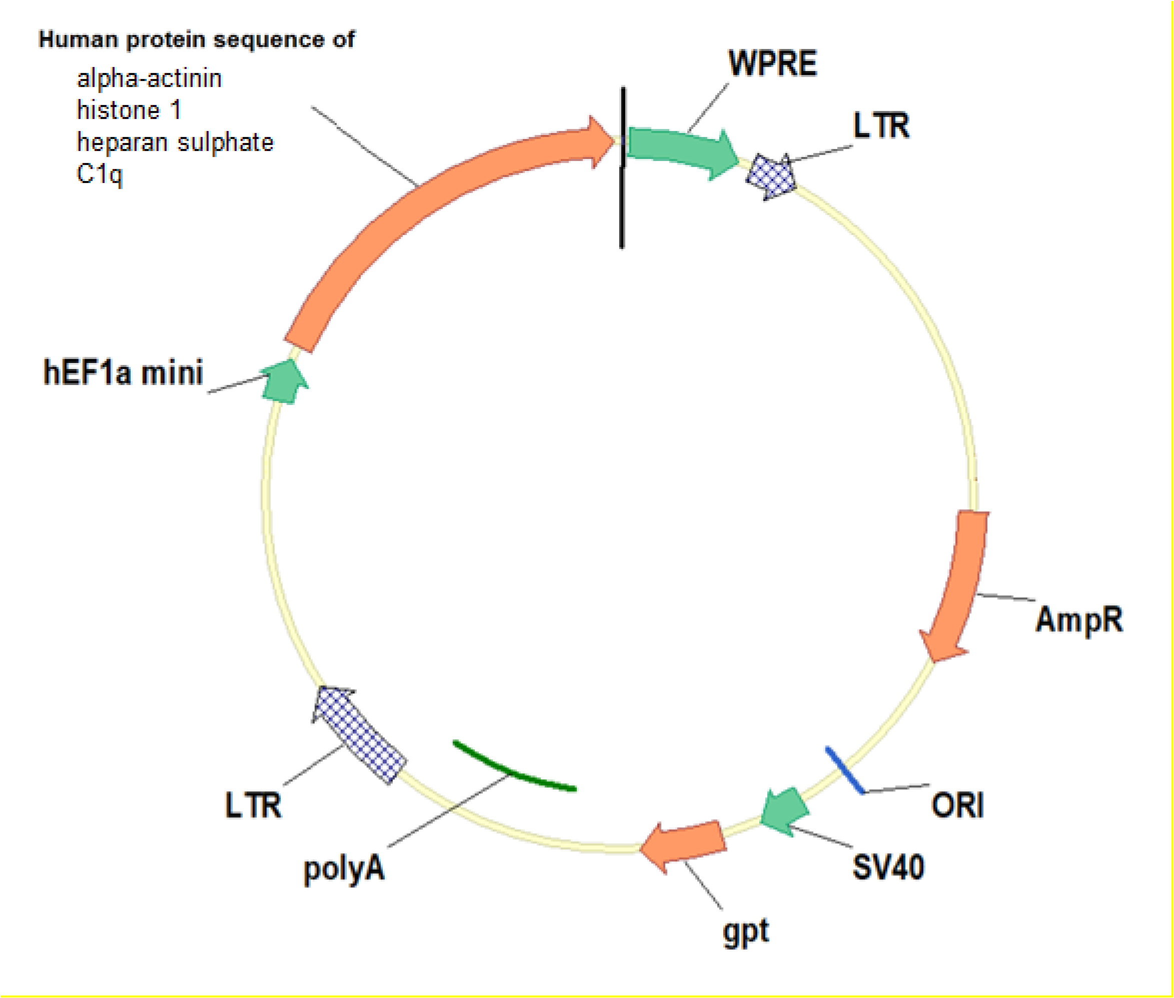

Supplementary Figure S2. **Quantification of anti-dsDNA antibodies in the culture medium of cultured isolated B cells.** B cells were isolated from healthy donors or anti-dsDNA⁻ lupus nephritis patients. B cells were non-stimulated or stimulated with INFβ (50ng/mL) during 48 hours. Anti-dsDNA titers were then measured in the culture medium by ELISA. In five independent experiments, no antibodies or low levels (less than 20 UI/mL) were detected.

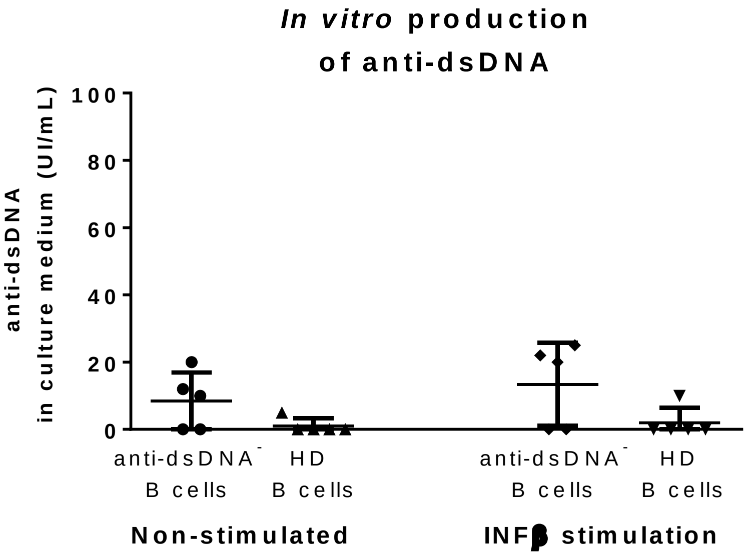

Supplementary Figure S3. Co-culture of control, DNA4 or DNA6-CAART with anti-dsDNA⁻ B cells. IFNγ secretion **(a)** and anti-dsDNA titers **(b)** were quantified using ELISA assay in the culture medium. No significantly differences were observed compared to control conditions (non-transduced T cells).

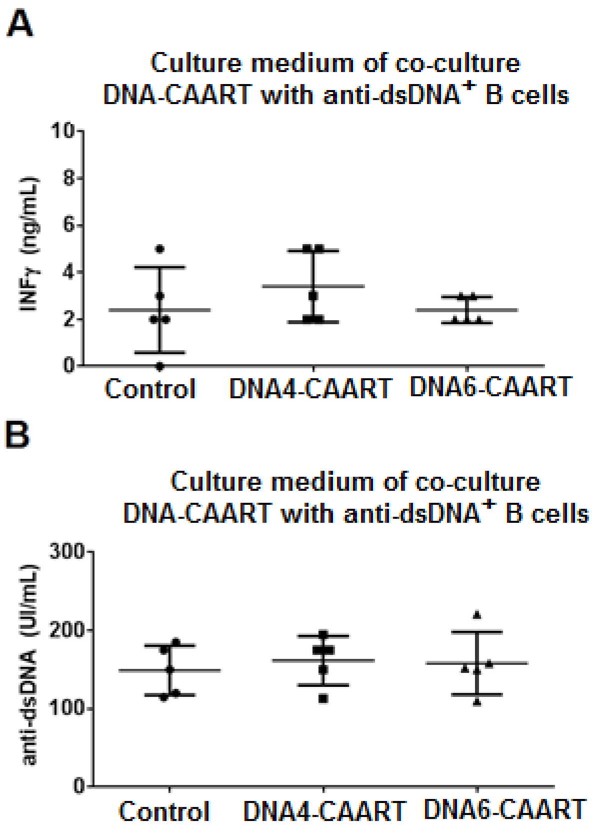

Supplementary Figure S4. Cytotoxicity of DNA4 and DN6-CAART in presence of high levels of anti-dsDNA in the culture medium. Using high concentration of dsDNA antibody standards (>1000 UI/mL) in the co-culture of DNA4 or DNA6-CAART with INF-α anti-dsDNA+ B cells (ratio 10:1, E:T), we observed a significantly decrease of cytotoxicity. The vertical axis shows the percentage of cytotoxicity, and the horizontal axis shows concentration of anti-dsDNA. Error bars represent the mean ± SEM from five experiments. Significant differences were calculated in comparison with non-presence of anti-dsDNA using paired t-test. ***p < 0.0001.

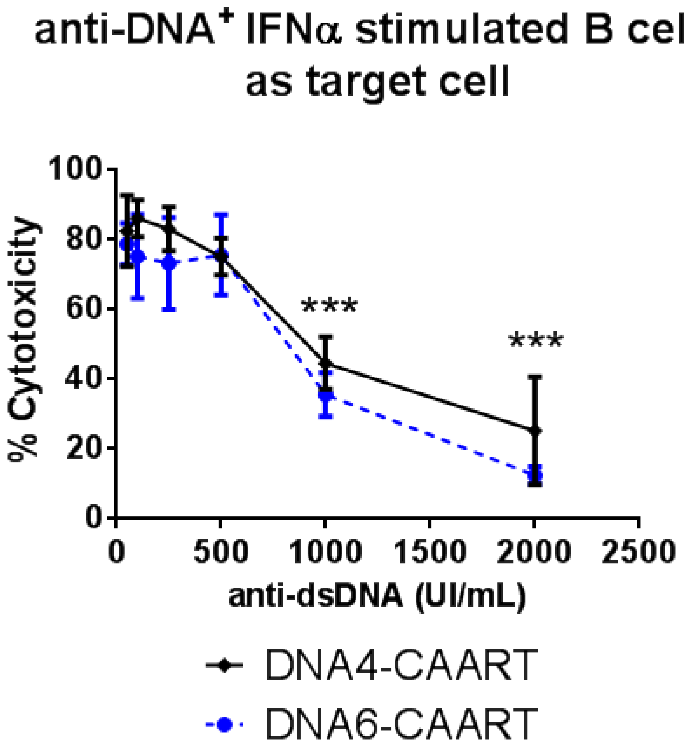

Supplementary Figure S5. Kidney organoid co-culture with B cells from healthy donor or anti-dsDNA⁻ LN patients. Study of kidney organoid morphology after co-culture with healthy donor or anti-dsDNA⁻ IFNα-stimulated B cells for 48 hours (n=5). Scale bar = 100µm. Statistical analysis was performed between groups using two-ways ANOVA. *p<0.05, **p<0.005.

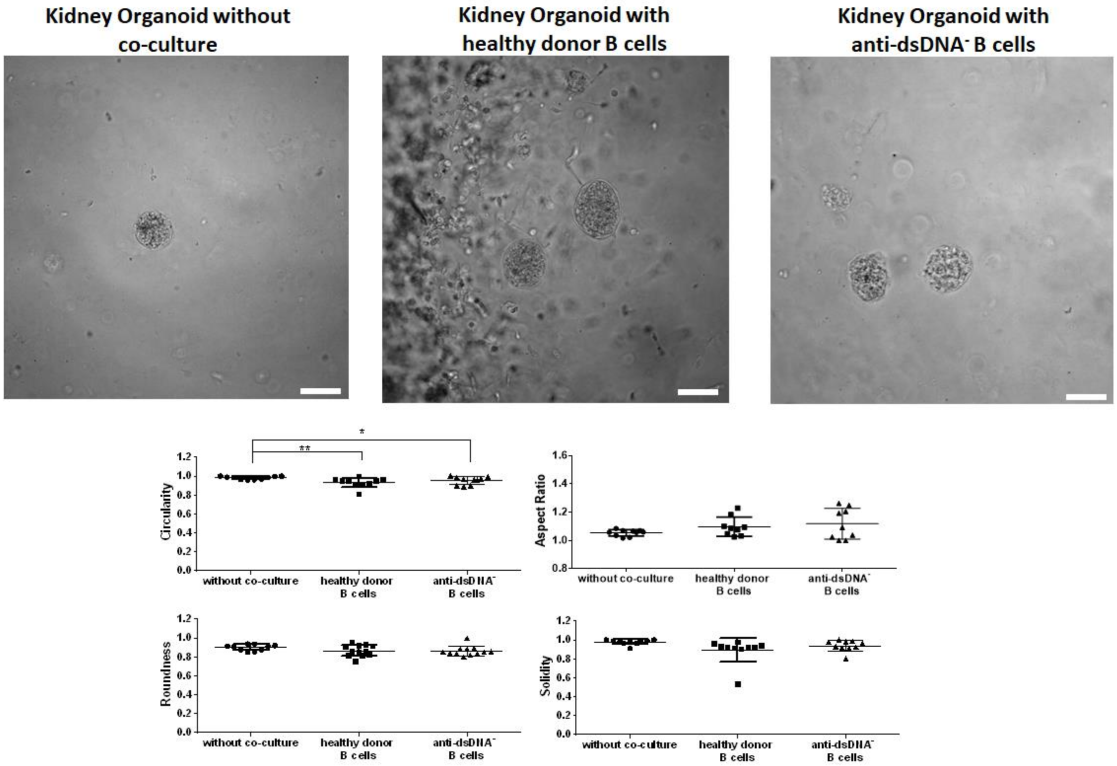

Supplementary Figure S6. Effect of control therapy using mock-transduced T cells on 3D immune-kidney organoid model damage. Morphology analysis of 3D immune-kidney organoid after 2-, 24- and 48-hours incubation with mock-transduced T cells as control treatment. Analysis of circularity, aspect ratio, roundness and solidity were performed using Image J software (n=5) and compared with DNA4-CAART treatment. Scale bar = 100µm. Statistical analysis was performed between groups using t-student in each time point. *p<0.05, **p<0.005, ***p<0.001.

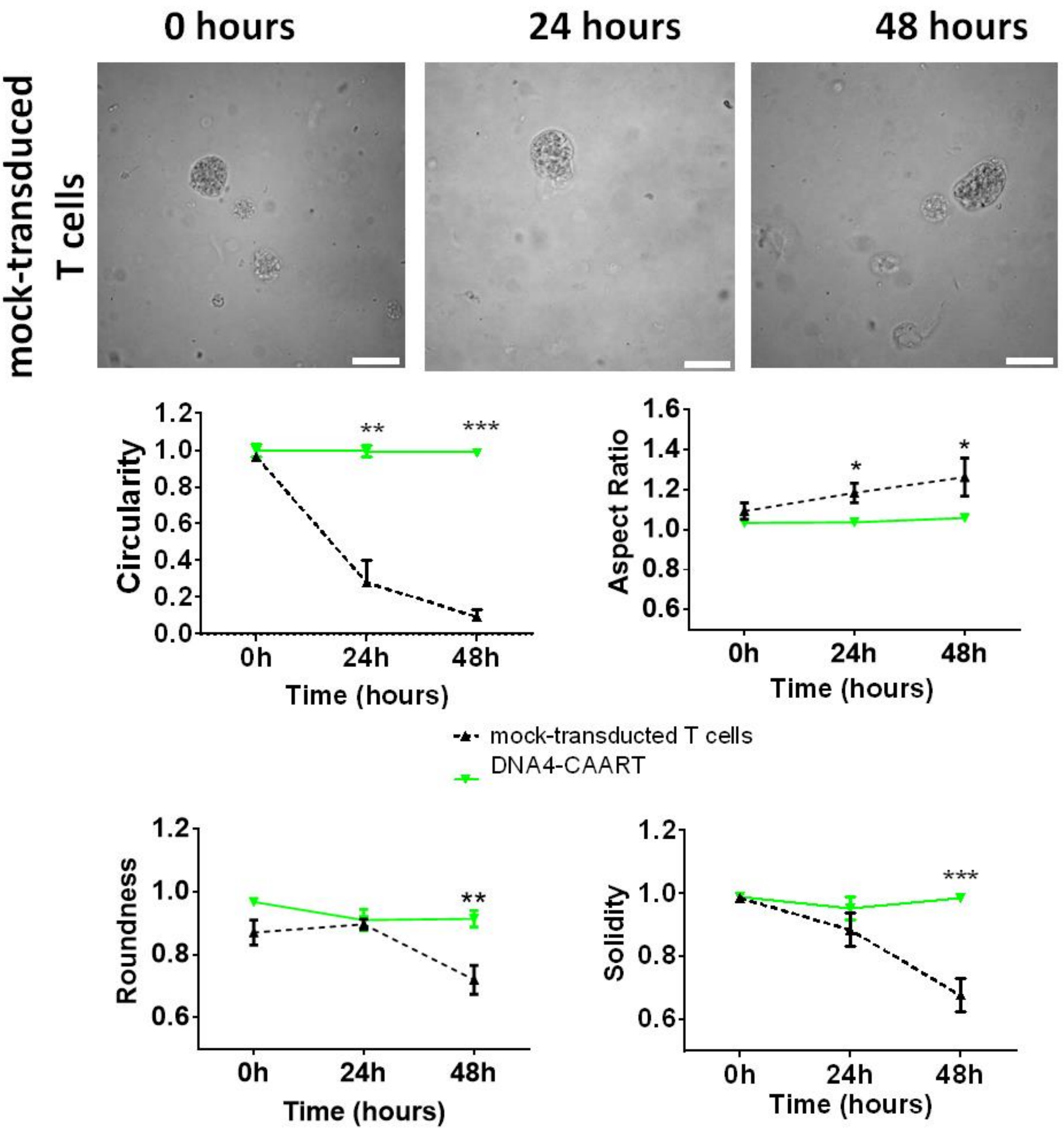

4. Supplementary Tables

Table S1. Antibodies used for cytometry and immunofluorescence staining.

Primary Antibody	Supplier	Code
**Anti-alpha actinin**	Genetex	GTX103240
**Anti-heparan sulfate**	Abcam	ab315240
**Anti-histone 1**	Genetex	GTX122561
**Anti-C1q (FITC)**	Genetex	GTX73479
**Anti-heparn sulfate proteoclycan 2 (FITC)**	CliniSciences	Orb8339
**Human CD19 (PE)**	Invitrogen	MHCD1904
**Secondary Antibody**	**Supplier**	**Code**
**Goat anti-rabbit IgG antibody (FITC)**	Genetex	GTX03116
**Alexa-488-conjugated anti-rabbit IgG**	Abcam	ab150077

Table S2. Primer IDs used in Taqman RT-qPCR from Applied Biosystems.

Gene	Assay ID (TaqMan)
**GADPH**	Hs02786624_g1
**ACTN1**	Hs00998095_m1
**HSPG2**	Hs01078507_m1
**HIST1H1A**	Hs00271225_s1
**C1QA**	Hs00706358_s1
**CASP3**	Hs00234387_m1
**BCL2L11**	Hs00708019_s1
**TP53**	Hs01034249_m1
